# Coverage of the 2019 Eastern equine encephalitis virus outbreak on news media

**DOI:** 10.34172/hpp.2020.43

**Published:** 2020-07-12

**Authors:** Corey H. Basch, Joseph Fera, Christie Jaime, Nasia Quininoes

**Affiliations:** ^1^Department of Public Health, William Paterson University, Wayne, NJ 07470, USA; ^2^Department of Mathematics, Lehman College, The City University of New York, New York City, USA

**Keywords:** Eastern equine encephalitis virus, Mass media, Disease outbreaks, Public health

## Abstract

**Background:** The media plays as an important role in delivering information about emergent issues, such as the Eastern equine encephalitis virus (EEEV) outbreak of 2019. As such, there has been an increase in news coverage of vector-borne disease coverage due to a rise in emerging or re-emerging arboviruses.

**Methods:** The purpose of this study was to describe the content of news clips related to the recent (2019) EEEV outbreak in the United States.

**Results:** Only 3 of the important topics identified were mentioned in a majority of the 110videos analyzed. These topics were, mosquito mentioned as transmitter, prevention by repellent/pesticide, and geography. Thus, many aspects of EEEV were lacking in coverage.

**Conclusion:** A priority for public health professionals should be to engage in discourse with newsmedia to assure that information disseminated via news channels is not vague or misleading.

## Introduction

Eastern equine encephalitis virus (EEEV) is a rare and potentially fatal *Alphavirus* , in the *Togaviridae* family.^1^ In the United States within the past year, nine states have reported 36 human cases (14 of them fatal) from the mosquito transmission of EEEV.^1^ Endemic cases of EEEV have been noted in the United States during the summer months, particularly as outdoor activities increase.^1^ The fact that humans are a reservoir as well as an amplification host for EEEV, increases concern.^2^ As per the Centers for Disease Control and Prevention (CDC), regulatory surveillance is conducted during peak seasons, with a focus on mosquito control for prevention.^1^ There is currently no vaccine or antiviral for treatment.^1^ Given the rare and unexpected nature in the rise of EEEV, it is natural for citizens to be concerned and to seek information. The media plays an important role in delivering information about such emergent issues.^3^ As such, there has been an increase in news coverage of vector-borne disease coverage due to a rise in “emerging or re-emerging arboviruses”.^4^ To date, there are no studies that describe the content of news media coverage of the recent EEEV outbreak, which is the purpose of this short communication.

## Methods

For the purpose of this cross-sectional study, as per the CDC, the current outbreak was defined as months in 2019 when cases of EEEV were reported. Thus, the sampling frame included videos of news clips spanning April to October 2019. Using the Google video function, all videos of news clips from the course of the 2019 EEEV outbreak in the United States to date were identified. This initial search included 823 videos. A total of 266 videos were excluded for being irrelevant (e.g. not a pertinent topic, photo only elicitations, etc.) and another 130 for being repeats. Coding was conducted on a subset of videos from the total sample, which was garnered by using systematic sampling (coding every nth video) which led to a final sample of 110. A coding sheet was adapted from a prior study of EEEV videos on YouTube,^5^ which was used to code news videos. Content categories are listed in Table 1. All videos were coded by one coder (NQ) and a second (CJ) coded a subset of 10 videos for inter-rater reliability, yielding a kappa of 0.91. This indicates a high level of agreement among raters. There were five prevention methods among the prescribed characteristics observed. Chi-square tests were completed to determine if video length or the gender of those featured in the video had an effect on the prevention methods mentioned. The data entry, organization, and analysis were conducted using MS Excel.

## Results

In total, 110 videos on EEEV were analyzed for this study. Descriptive statistics were run for video length, the gender of those featured in the video, and on whether or not the video included prescribed characteristics. In some cases, the length, gender information, and characteristic data was not accounted for. Thus, some counts do not total to 110.

Of the 110 videos analyzed, their lengths ranged from 8 seconds to 565 seconds (9 minutes, 25 seconds) with an average length of 1 minute, 52 seconds. In total, 28 videos were less than 1 minute, just under half (47) were between 1 and 2 minutes in length, 23 were between 2 and minutes, and 11 were more than 3 minutes long.

Roughly 36% (95% CI: 27%, 45%) of the videos had information provided by a woman only, while only about 9% (95% CI: 4%, 14%) had information provided by a man only. Almost 41% (95% CI: 32%, 50%) of the videos had both a man and a woman giving information, while just under 13% (95% CI: 7%, 19%) of the videos featured no people.

A list of topics is shown in Table 1 along with an indication of how many of the videos mentioned this topic (yes) and how many did not mention this topic (no). Of the 25 topics included, only 3 of them were mentioned in a majority of the videos analyzed. These topics were, mosquito mentioned as transmitter, prevention by repellent/pesticide, and geography – where it is most possible. The death of a human was mentioned in 54 total videos which is just over 49%.

Table 2 shows the *P* values resulting from the chi-square tests performed to determine whether video length and/or the gender of those featured on the video impacted the prevention methods mentioned in the video. The analysis considered the effect significant for *P* <0.05. Interestingly, both length and gender had a significant impact on whether the prevention methods of repellent/pesticides and staying in doors were mentioned. Neither length nor gender, however, played a role in whether or not the other three prevention methods were mentioned.

## Discussion

The results of this study indicate that videos of news coverage of EEEV often leave out essential information about symptoms, highlight the worst-case scenario, and underplay the steps that can be taken to mitigate risk. The processing and understanding of health news can play a major role in how members of a community react or take action during a public health crisis. For example, coverage of the 2016 Zika virus outbreak brought to light the possibility for trending media that increased confusion and provoked fear.^6^ Inaccurate, incomplete, or misinformation in media can set into motion a reaction of pandemonium.^7^ As was the case with coverage of the Ebola virus, media often lacks focus on preparedness and prevention.^8^

This study is limited by the sampling frame, which consists exclusively of videos housed on the internet, searchable by the Google news function. One can presume that there are a multitude of additional news broadcasts pertaining to the recent EEEV outbreak that have not been posted to the internet. Because the EEEV outbreak in the US 2019 was so geographically specific, Google news videos provide an assortment of local coverage from affected areas. As with any cross-sectional study, this information cannot be generalized to a larger audience. Additionally, the tools used in this study did not allow for tracking the number of views, which is helpful in knowing the far-reaching effects of messaging.

## Conclusion

A priority for public health professionals should be to engage in discourse with news media to assure that information disseminated via news channels is not biased, vague, or misleading, especially during times of outbreaks of emerging infectious diseases, such as EEEV. Future interventions related to EEEV could include media as a potential partner in an effort to educate the public about awareness and prevention.

## Ethical approval

The IRB at William Paterson University does not review studies in which human subjects are not involved.

## Competing interests

None of the authors report a conflict of interest.

## Authors’ contributions

CHB and CJ conceptualized the study. NQ collected the data, which was then analyzed by JF. All authors contributed to manuscript production.

**Table 1:** A list of topics followed by the count of how many of the videos analyzed mentioned this topic (yes) and how many did not (no)

**Topic mentioned/showed in video**	**Yes**	**No**
Mosquito as transmitter	99	11
Pain in humans	2	108
Anxiety/fear of diagnosis/screening	23	87
Horse as transmitter	18	92
Fatigue symptoms in humans	20	89
Headache symptoms in humans	25	83
Fever symptoms in humans	32	78
Treatment in humans	1	109
Chills symptoms in humans	15	95
Vomit symptoms in humans	8	102
Swollen brain in human	27	83
Damage to heart	0	110
Prevention: clothing	40	70
Prevention: repellent/pesticides	71	39
Prevention: staying in doors	43	67
Prevention: removal of habitat	16	94
Prevention: vaccination of pets	14	96
Selling a product or service	1	109
Geography: where it is most possible	95	15
Other types of encephalitis	12	98
About an individual experience	21	89
Death of a human	54	56
Death of a horse	7	103
Death of another animal	2	108
Only about horses, no humans	1	109

**Table 2:** The results of the chi-square tests done to determine if video length or the gender of those featured on the video effected prevention methods mentioned

**Prevention mentioned**	**Length** ***P*** v**alue**	**Gender** ***P*** v**alue**
Clothing	0.0547	0.1949
Repellent/pesticide	0.0000	0.0094
Staying indoors	0.0100	0.0055
Removal of habitat	0.4236	0.4672
Vaccination in pets	0.1601	0.2660
